# Exceptional Extension of Benign Angiomyolipoma in the Renal Vein

**DOI:** 10.5334/jbsr.2854

**Published:** 2022-09-22

**Authors:** Darius Lepot, Patrick Mailleux, Stéphane B. Van den Broeck

**Affiliations:** 1 UCLouvain, BE; 2 Clinique Saint-Luc Bouge, BE

**Keywords:** angiomyolipoma, renal vein extension, renal neoplasm, abdominal imaging, unenhanced CT

## Abstract

**Teaching Point:** When a renal angiomyolipoma (AML) is incidentally detected on imaging, the venous system should be assessed for intravascular fat component.

## Case History

**Figure 1:**
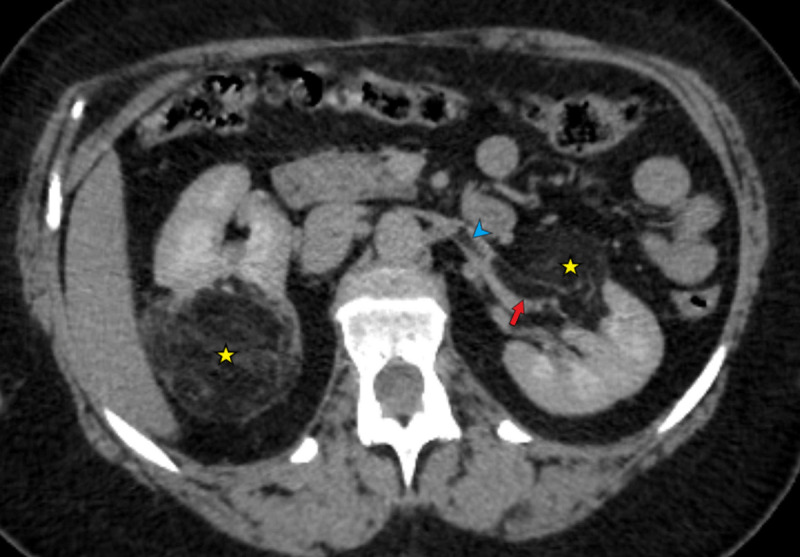


**Figure 2:**
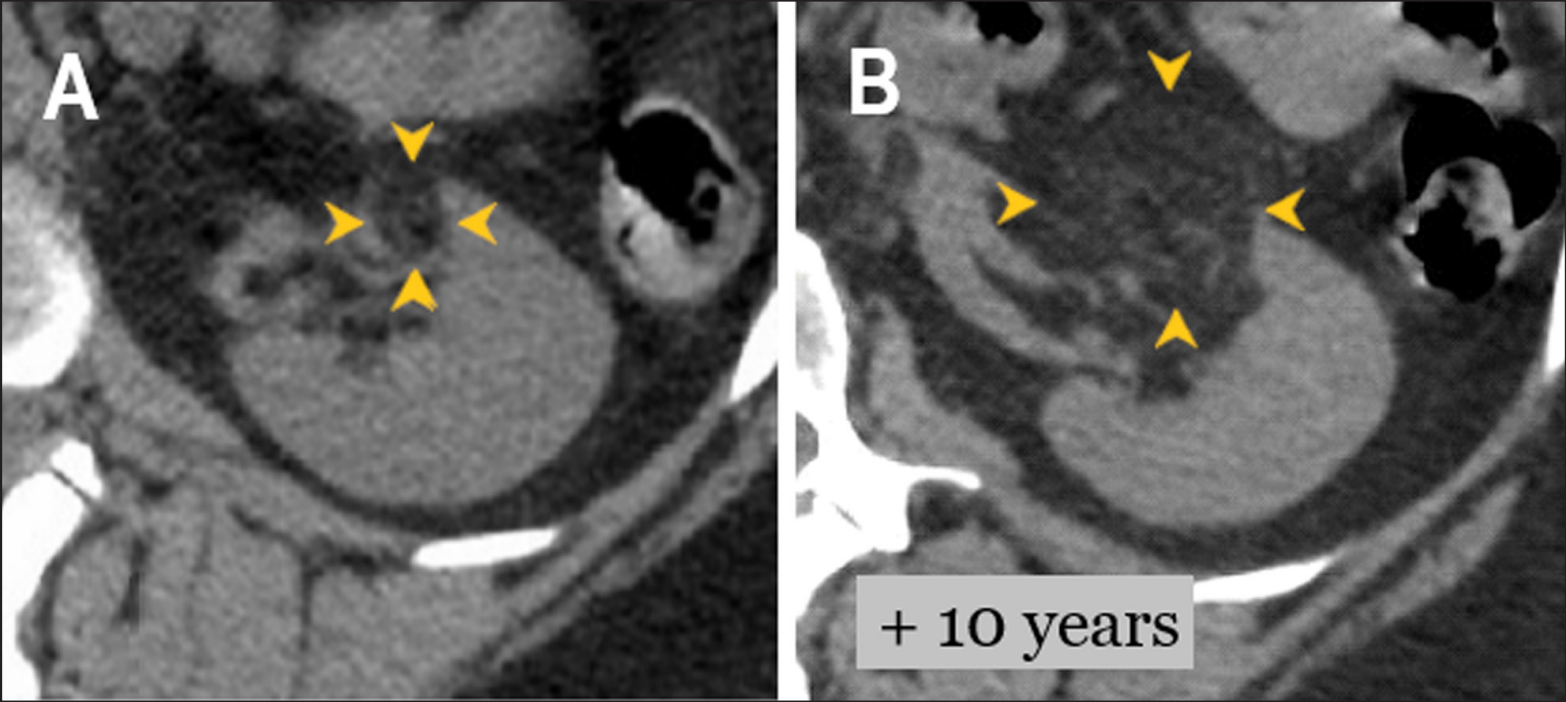


A 61-year-old female was admitted to the emergency department for epigastric pain following epigastric hernia surgery. An abdominal unenhanced computed tomography (CT) demonstrated a reoccurrence of epigastric eventration. As an incidental finding, two round well-defined lesions of fat density (–78 HU) containing tortuous foci of enhancing vessels features strongly suggestive of renal AMLs (asterisk in Figure 1). Both AMLs showed a fourfold increase in size to prior imaging 10 years ago; the right posterior cortical AML measured 6.6 cm and left anterior hilar AML measured 3.2 cm (arrowheads in Figure 2).

**Figure 3:**
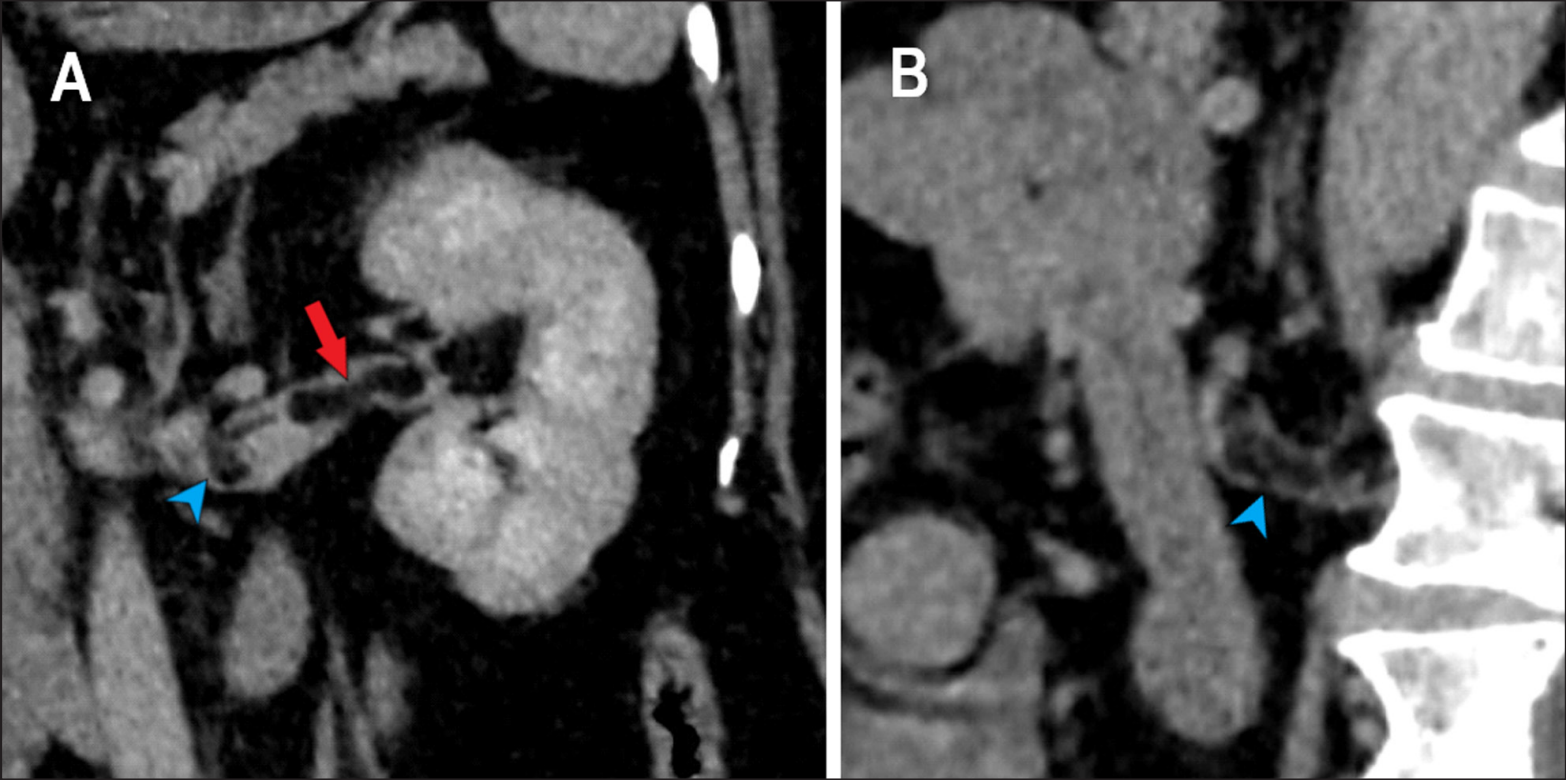


The lipomatous component unexpectedly extended into the left renal vein, forming one 4.5 cm fat density intravascular flap (arrows on Figures 1 and 3A) and a second, smaller, 2 cm intravascular fat density lesion located more proximally, in a tributary vein (arrowheads in Figures 1 and 3). A contrast-enhanced CT outlined the intravenous extension more clearly, showed no extension into the inferior vena cava, and excluded pulmonary embolism (PE).

Total left nephrectomy and right partial nephrectomy were planned. Histologic examination confirmed the diagnosis of AML without any evidence of sarcomatoid dedifferentiation.

## Comment

AML is a common asymptomatic benign renal neoplasm mostly detected incidentally during abdominal cross-sectional imaging. It is composed of thick-walled blood vessels, smooth muscle, and mature adipose tissue. In 20% of cases there will be an association with tuberous sclerosis manifesting as multifocal and bilateral AMLs. A common symptomatic presentation occurring with large AMLs (>4 cm) and leading to surgical treatment or embolization is spontaneous retroperitoneal hemorrhage sometimes complicated by hypotensive shock.

A rare case of AML showing liposarcomatous transformation has also been reported. Aggressive patterns with intravascular growth is a rare condition only described in a couple of case reports [1]. Mostly the extension occurs in the renal veins and inferior vena cava (IVC). Extension of a lipomatous thrombus in a renal vein tributary, as in our case, has not yet been described. The increased risk for PE emphasizes the need for surgery and sometimes for IVC filter placement [2].

Even a benign, and relatively frequently encountered, AML can rarely exhibit aggressive behavior, with intravascular potentially life-threatening fat extension. The radiologist should pay attention to this and attentively review the renal veins and IVC for a fat density component that suggests intravascular extension or embolization [3]. Therapeutic options, that is, potentially lifesaving surgery must be discussed with the patient.
